# Practice of debriefing of critical events: a survey-based cross-sectional study of Portuguese anesthesiologists

**DOI:** 10.1097/j.pbj.0000000000000215

**Published:** 2023-06-23

**Authors:** Daniel Teles, Mariana Silva, Joana Berger-Estilita, Helder Pereira

**Affiliations:** a Department of Anaesthesiology, University Hospital Centre of São João, Porto, Portugal; b Institute for Medical Education, University of Bern, Bern, Switzerland; c CINTESIS—Centre for Health Technology and Services Research, Faculty of Medicine, Porto, Portugal; d Surgery and Physiology Department, Faculty of Medicine, University of Porto, Porto, Portugal

**Keywords:** debriefing, critical event, anesthesia, health care CRM, Portugal

## Abstract

Debriefing is an essential procedure for identifying medical errors, improving communication, reviewing team performance, and providing emotional support after a critical event. This study aimed to describe the current practice and limitations of debriefing and gauge opinions on the best timing, effectiveness, need for training, use of established format, and expected goals of debriefing among Portuguese anesthesiologists.

**Methods:**

We performed a national cross-sectional online survey exploring the practice of anesthesiologists' debriefing practice after critical events in Portuguese hospitals. The questionnaire was distributed using a snowball sampling technique from July to September 2021. Data were descriptively and comparatively analyzed.

**Results:**

We had replies from 186 anesthesiologists (11.3% of the Portuguese pool). Acute respiratory event was the most reported type of critical event (96%). Debriefing occurred rarely or never in 53% of cases, 59% of respondents needed more training in debriefing, and only 4% reported having specific tools in their institutions to carry it out. There was no statistical association between having a debriefing protocol and the occurrence of critical events (*P*=.474) or having trained personnel (*P*=.95). The existence of protocols was associated with lower frequencies of debriefing (*P*=.017).

**Conclusions:**

Portuguese anesthesiologists know that debriefing is an essential process that increases patient safety, but among those surveyed, there is a need for an adequate debriefing culture or practice.

**Trial registration:**

Research registry 7741 (https://www.researchregistry.com/browse-the-registry#home).

## Abstract 2

Supplemental Digital Content is Available in the Text.

## Introduction

Critical events are common in anesthesia practice and can be a significant source of stress for physicians. The estimated incidence of critical events is 145 per every 10,000 cases.^1^ In Portugal, this may represent more than 10,000 critical events per year.^2^

Postcritical event debriefing is held in clinical settings among health care providers as an education, team learning, and patient safety intervention.^3-5^ Through a guided learning conversation based on mutual reflection of clinical practice, participants explore the relationships among events, processes, and performance outcomes of a clinical situation.^6,7^ There are two types of debriefing: the “hot” and the “cold” debrief. Hot debriefing is performed immediately after the critical event.^8,9^ It has the advantage of an earlier intervention, improved participation, and improved recall of events. These hot debriefs often occur in patient care areas, including all key players during the critical event and are voluntary, timed, and facilitated by any team member.^8,9^ On the other hand, a “cold debrief” is a delayed debrief that occurs days or weeks after a clinical event. Advantages of cold debriefs include having more time to collect data, additional staff or expert facilitators may be invited to attend, and staff have had a chance to reflect. However, compared with “hot debriefs,” the events may not be recalled by participants. Some participants may not be able to attend, immediate support for staff is not provided, and learning from the event may be delayed. Cold debriefs are best viewed as complementary to hot debriefs and may be informed by the hot debrief.

A large body of literature demonstrates the benefits of debriefing after critical events. Debriefing offers a health care team the opportunity to re-examine the clinical encounter, discuss individual and team performance, identify errors, and develop performance improvement strategies through reflective learning processes.^10,11^ Although real-time clinical event debriefing can be challenging to implement, it has been identified as an essential aspect of effective clinical education, quality improvement, and learning systems.^10,11^ Debriefing can also help protect and support those exposed to critical incidents by minimizing abnormal stress responses and the second victim effect.^12^ Added to its effects on team performance, debriefing as a factor in improving patient outcomes is a significant area of research.^13^ Unfortunately, few institutions both in Portugal and elsewhere have formal guidelines and standards on team debriefing after critical events.^14,15^ In addition, anesthesiologists have limited data regarding debriefing practice after critical events.^16^

This study aimed to describe the current practice and limitations of debriefing and gauge opinions on the best timing, effectiveness, need for training, use of established format, and expected goals of debriefing among Portuguese anesthesiologists.

## Methods

### Ethics

The Institutional Review Board review at the Ethics Committee of the S. Joao University Hospital Centre (Nr. 114-22) waived the need for ethics approval. The survey link included a cover letter reiterating the study's goals and that participation in the survey was considered “consent by participation.” Owing to the expected small sample size, we used ID numbers to code participants and requested no directly identifying data. Data were stored in a secure repository accessible to the investigators only. As applicable, all procedures from this investigation followed the Declaration of Helsinki. All researchers complied with the Data Protection Acts of their respective academic institutions.

### Study design and setting

This cross-sectional online survey-based study was aimed at active anesthesiologists (in practice and training) in Portugal.

#### Primary and secondary outcomes

The primary outcome of this study was the summative overview of witnessed events. Secondary outcomes included the analysis of the need for debriefing in relation to a reported event, and the current practice of debriefing after a critical event was collected, including information on who lead debriefing sessions, how often, how effective, how soon or frequent, and what happens during debriefing sessions. Demographic information of each participant (position and years of clinical experience) was obtained. We also asked participants whether they had had any prior training, whether there was a need for training, and what kind of events should be debriefed. We were also interested to know whether they had any established format, whether they felt debriefing was necessary, and about their perceived goals and barriers to performing debriefing in their various departments.

#### Survey development

We developed the questionnaire based on a previous publication by Arriaga et al.^17^ The different aspects formulated in the primary and secondary outcomes were reflected in the survey items. Participants were queried for the occurrence of critical events, including operating room crises and disruptive behavior that undermined a culture of safety. In addition, participants were asked about their current position, exposure to critical events, current practice, and opinion regarding the debriefing process, namely the structure and objectives that it should comprise. A 20-item questionnaire was developed in Portuguese (Supplementary Digital File 1, Supplementary Digital Content, http://links.lww.com/PBJ/A30, and Supplementary Digital File 2, Supplementary Digital Content, http://links.lww.com/PBJ/A31: Portuguese and English Versions of the Questionnaire): eleven questions were multiple choice and nine questions required the use of a 5-point Likert scale from 1 (I totally disagree) to 5 (I totally agree). All items were optional. All questions were aimed at participants working in a primary unit (i.e., department). The draft questionnaire was piloted by six clinicians (trainees and consultants) from two different institutions. The usability and technical functionality of the electronic questionnaire was tested before fielding. The questionnaire was hosted online at Google Forms (Google, Mountain View, CA). The questionnaire used in this study was anonymous and did not collect identifiable information such as names, addresses, or contact details. To prevent multiple replies from the same responders, we set Google Forms® to use a unique identifier code for each participant to ensure that each respondent could only complete the survey once. We also used built-in software to detect and remove any duplicate or fraudulent responses. The corresponding database was generated by an automatic embedded method for capturing responses. Respondents could review and change their answers before submitting their replies. No incentives were offered.

#### Survey distribution and sample size calculation

As the primary mode of survey distribution was through social media using the snowballing sampling technique,^18^ selection bias was reduced by aiming to collect at least 10% of the national society's members.^19^ The survey link was distributed in July, August, and September 2021, with a dissemination of the questionnaire through e-mail and telephone contacts of active Portuguese anesthesiologists. We performed an open survey with a convenience sample.

### Statistical methods

Quantitative data were analyzed with SPSS v26 (IBM, NY). We performed a descriptive analysis of the survey data. Categorical variables were described as absolute (n), relative frequencies (%), and continuous variables as mean ± SD. Individual survey items were assessed for normal distribution with the Shapiro-Wilks test and visual assessment of residuals and Q-Q Plots. Owing to the nature of the data, we used the chi-square test or Fisher exact test for categorical variables. An a priori probability of less than 0.05 was considered statistically significant. For reliability testing of the checklist, internal consistency was evaluated with Chronbach alpha.

## Results

One hundred eighty-six anesthesiologists participated in this study, 76 (41%) trainees and 110 (59%) consultants. According to the 2017 Census,^20^ this corresponds to 11.3% of the anesthesiologists pool in Portugal. In total, participants described 1285 critical events during their practice. Table 1 presents the current position of anesthesiologists who responded to the survey and the typology of critical events that were witnessed. All replies were analyzed, including the incomplete surveys.

**Table 1:** Participants' demographics and types of critical events witnessed, n (%)

n (%)	Trainees (n=76)	Consultants (n=110)	Total number of anesthesiologists (n=186)	*P* value
Current position				
Trainee 1^st^/2^nd^ year	30 (16)	—		—
Trainee 3^rd^/4^th^ year	29 (16)	—		—
Trainee 5th year	17 (9)	—		—
Consultant <5 years	—	34 (18)		—
Consultant >5 years	—	76 (41)		—
Type of event witnessed (n=1285)				
Perioperative death	41 (54)	99 (90)	140 (75)	<.001
Cardiopulmonary resuscitation	35 (46)	99 (90)	134 (72)	<.001
Malignant hyperthermia	2 (3)	12 (11)	14 (8)	<.001
Arrhythmia with hemodynamic instability	46 (61)	104 (95)	150 (82)	<.001
Acute respiratory event	71 (93)	107 (97)	178 (98)	<.001
Need for surgical airway	9 (12)	38 (35)	47 (25)	<.001
Anaphylaxis	26 (34)	68 (62)	94 (51)	<.001
Toxicity of local anesthetics	4 (5)	23 (21)	27 (15)	<.001
Transfusion reaction	23 (30)	25 (23)	48 (26)	N/S
High spinal block	15 (18)	41 (37)	55 (30)	<.001
Medication error	29 (38)	83 (75)	112 (60)	<.001
Fire/electrical hazard	3 (4)	10 (9)	13 (7)	<.001
Ventilator failure	21 (30)	69 (63)	92 (49)	<.001
Disruptive behavior	12 (20)	48 (44)	63 (34)	<.001

N/S, nonsignificant.

The most reported critical event (witnessed by 98% of participants) was “acute respiratory event,” followed by arrhythmia with hemodynamic instability and cardiopulmonary resuscitation. Consultants witnessed significantly more types of events when compared with trainees (*P*<.001).

### Patterns of debriefing

Debriefing was reported to occur rarely or never in 53% of cases, with only 10% being performed almost always or always. Consultants significantly reported higher frequencies of performing debriefing when compared with trainees (*P*=.004). Most respondents (62%) identified that the adequate time for debriefing was immediately after the event/on the same day. Consultants significantly reported higher performance of debriefing immediately after the critical event/on the same day when compared with trainees (*P*<.001). However, 67% of respondents reported having felt the need and suggested a debriefing, which did not occur. This was not different between trainees and consultants (*P*=.333). In addition, qualitative thematic descriptions of the goals and barriers to debriefing in our sample are presented in Table 2.

**Table 2:** Goals and barriers for debriefing (n=186 responders). Values are for the number of replies, and percentages are for the proportion of responders.

	Total, n (%)
Goals	
Develop plans to improve performance at an upcoming event	180 (97)
Review process and system failures	168 (90)
Discuss team performance	160 (86)
Reviewing medical attitudes	149 (80)
Discuss the psychological impact and provide emotional support	142 (76)
Discuss the individual performance of the people involved	62 (33)
Other	3 (2)
Barriers	
The workload does not allow the time needed	99 (53)
No interest from colleagues in general	94 (51)
Feeling criticized/judged	78 (42)
Communication difficulties (during the debriefing)	68 (37)
Not having the necessary training	57 (31)
No debriefing tools available	44 (24)
No suitable place available	44 (24)
Other	4 (2)

### Training

When asked about specific training, 59% of respondents reported never having had any training, and only 4% reported having specific tools in their institutions to perform debriefing. Table 3 presents the mean scores of each questionnaire item. Consultants reported having more training in debriefing (*P*=.003). No other statistically significant differences between consultants and trainees were noted.

**Table 3:** Analysis of the Likert scale questionnaire. Comparisons between trainees' and consultants' mean values for each answer. Data displayed in mean ± SD

Item	Statement	Trainees (n=76)	Consultants (n=110)	Total (n=186)	*P* value
1	I consider debriefing an important tool	4.83±0.67	4.82±0.53	4.83±0.55	.368
2	Debriefing increases patient safety and contributes to outcome improvement	4.79±0.72	4.77±0.59	4.78±0.61	.384
3	The most senior doctor must establish the content of the debriefing	3.00±0.13	3.25±0.13	3.17±1.23	.053
4	Debriefing must be guided by someone not involved in the critical event	3.19±0.13	3.17±0.13	3.17±1.26	.061
5	Participants in debriefing should be impartial	3.88±0.12	4.00±0.12	3.96±1.15	.148
6	The debriefing of events with adverse outcomes are more than that of events with a successful outcome	2.39±0.14	2.32±0.13	2.37±1.33	.346
7	I consider that my institution has a proper culture of debriefing	1.96±0.12	1.95±0.96	1.96±1.03	.920
8	I consider having the adequate training to perform a debriefing	2.27±0.13	2.89±0.12	2.63±1.17	.003
9	I consider that there is a need for training in debriefing in my institution	4.35±0.09	4.30±0.08	4.37±0.86	.795

There was no statistical association between having a debriefing protocol and the occurrence of critical events (*P*=.474) or having trained personnel (*P*=.950). There was a statistically significant association between the inexistence of protocols and reports that there is no culture of debriefing in the institution (*P*=.014). Participants working in institutions that do not have debriefing protocols report having less adequate training to perform debriefing (*P*=.032). The existence of protocols was associated with lower frequencies of debriefing (*P*=.017). The Chronbach alpha for items 13 to 20 was 0.652, which is weak. No exclusion will increase Chronbach alpha, so no items need to be revised or excluded.

## Discussion

This is the first study describing current practices of debriefing of Portuguese anesthesiologists. In our cohort, debriefing was reported as absent or rarely performed in over half of the reported cases. This is in line with current international literature. After an unexpected death in the operating room, Canadian anesthesiologists reported a 14% debriefing rate.^9^ Among anesthesiology trainees in Canada, 36% had never participated in a debriefing.^16^ Another study including pediatric emergency physicians in the United States reported that 30% of respondents had never participated in a debriefing session after medical resuscitation events.^21^ In a study of critical events experienced by anesthesiology trainees at a large academic medical center over one year, only 49% of the events were associated with at least some bare minimum components of a proximal debriefing that included the study participant.^17^ Although most studies fail to give specific reasons for this lack of debriefing, themes from free-text participant comments called for formal policies and encouragement for support individualized to specific debriefing needs.

Our cohort mentioned the unavailability of dedicated time for debriefing and other contributing factors, such as lack of trained facilitators, fear of judgment by peers, general discomfort with the event, lack of administrative support, and lack of adherence to a debriefing. Several studies mention barriers to carrying out debriefing. These include too many urgent patient care issues, lack of trained debriefing facilitators, fear of judgment from colleagues, discomfort regarding the event, lack of administrative support, and overall buy-in.^11,21,22^ We hypothesize that the constant struggle with constraints of production pressure, coupled with limited time and space, in busy surgical and procedural areas may be generalized to different specialties and parts of the world.

Consultants performed debriefing more frequently and more adequately than trainees. Although this result is not surprising, it does mirror the lack of debriefing training in the early postgraduate years. Despite the vulnerable position in which trainees find themselves—at the front lines of care with far less experience to draw from than their attending counterparts—most training programs provide little training for preparation for critical events before their occurrence. Courses on debriefing are usually offered to more senior specialists, who often take the leading role in a critical event.^23^ However, this stance may not reflect an increase in debriefing quality, as debriefing is challenging to learn. While there is a paucity of studies on peer debriefing in anesthesiology, data from other fields demonstrate that instructor-led debriefing seems to be of higher quality and brings about increased skills compared with peer debriefing.^24^ Other studies indicate that peer-facilitated and faculty-facilitated debriefing is equally effective.^25^ We hypothesize that peer debriefing may be advantageous and should be offered during training. Trainees have been shown to seek their peers to talk about their experiences with recent critical events.^26^

Most responders agreed that debriefing should be performed immediately after the critical event. This so-called hot debriefing is the preferred choice for formal debriefing immediately postevent.^8,9^ The principal advantage of an earlier intervention is an improved recall of events. There is no evidence of one type of debriefing being more beneficial than the other. However, “hot” debriefs seem to be preferred to cold debriefs in clinical settings.

More than half of the respondents reported having felt the need and suggested doing a debriefing, but it did not occur. This finding emphasizes the known current gap between the well-established positive role of debriefing in the literature and the actual clinical practice.^27^ Although debriefing seems to be rarely performed after a critical event, health care workers generally feel that, in such situations, debriefing should always occur.^21^ Health care workers from distressing environments agree that debriefing is an essential tool that increases patient safety and improves patient outcomes, mainly when performed as a standard practice.^7^ Furthermore, there is a strong belief in the positive future consequences for those involved in the debriefing process, either through the improvement of technical skills or through the confidence to connect with similar situations in the future.^28,29^ Despite that, there is an apparent lack of adherence to the debriefing process, which points to an implementation issue within the organizations. Some described strategies to improve compliance with debriefing include central hospital-wide departmental support, efficient documentation methods, and highly motivated groups of professionals involved in implementing a debriefing tool as part of their quality improvement project.^27^ Therefore, creating a working environment that encourages professionals to use debriefing is a fundamental step in enhancing debriefing compliance. The mere standardization of the debriefing session can mitigate potential iatrogenic emotional effects of the debriefing and professionals' burnout.^30^ In this context, organizations should define the minimum types of events to be targeted and other critical elements of debriefing. In particular, the debriefing format model that fits the institution's reality, coupled with specific training sessions and regular encouragement, makes debriefing an integral part of the work process.

Our results also indicate that most health care providers have never received any form of training on debriefing and strongly agree with the need for such training. This has been observed in other studies.^31^ In addition, respondents reported that there was no format for debriefing in their departments and that they would prefer to use one. During debriefing, a structured approach serves as a guide that allows conversations to unfold systematically, keeps the discussion on track, assists an efficient use of time, and focuses on the relevant learning goals.^29^ All in all, our study may indicate that there is a lack of a formalized debriefing culture within Portuguese health care institutions. Debriefing may not be prioritized or valued as highly as other aspects of patient care, such as technical skills or clinical outcomes.

Our results should be interpreted in the context of the study design and its limitations. Our cross-sectional study is subject to nonresponse bias; participants could opt out of the study, which can create a bias. There is also a possibility of a recall bias, as respondents were asked to recall their debriefing practice over an unspecified period. We also did not previously validate the questionnaire, and the assessment of reliability should have been performed by verifying both internal and external consistency. We had a limited number of respondents, and it was performed within a specific country, which may impair generalizability. Although our findings align with previous studies, a larger study will be able to portray these practices with increased power. Moreover, we were unable to determine the association of the practice of debriefing with hospital size. These aspects may be addressed with future studies. Finally, there is a possibility of a volunteer bias, as the people who agreed to participate may not represent the entire population.

## Conclusions

We report for the first time a perspective on debriefing practices among Portuguese anesthesiologists and highlight areas for potential improvement within this population. In our cohort, debriefing was reported as absent or rarely performed in over half of the reported cases. While responders agreed that debriefing should be performed immediately after the critical event, in practice, there was little time reported for debriefing during daily practice. Our findings mirror the current need for customized tools adapted to local practice. Our findings may serve as a basis for further exploration of debriefing practices within similar contexts. Given the broad potential impact of critical events on patients, professionals, and health care systems, continued research into viable, generalizable, and sustainable interventions for proximal support after these events are critical.

## Conflicts of interest

J. Berger-Estilita is an associate editor for BMC Medical Education and has received travel expenses from Medtronic for the *Save the Brain Initiative* training. The remaining authors declare that they have no known competing financial interests or personal relationships that could have appeared to influence the work reported in this paper.

## Availability of data and materials

All data generated or analyzed during this study are included in this published article and its supplementary information files.

## Authors’ contributions

DT and HP recruited the participants. DT, MS, and HP had the idea for the study and conceptualized its methodology. DT and MS performed the data collection and analysis. DT, HP, and JBE interpreted the analysis and drafted the paper. All of the authors critically revised the manuscript for important intellectual content and agreed to submit the final version of the manuscript for publication.
